# Longitudinal development of the dust microbiome in a newly opened Norwegian kindergarten

**DOI:** 10.1186/s40168-018-0553-x

**Published:** 2018-09-15

**Authors:** Anders B. Nygaard, Colin Charnock

**Affiliations:** 1 Disease and Environmental Exposures Research Group, Department of Life Sciences and Health, OsloMet - Oslo Metropolitan University (OsloMet), Oslo, Norway; 2 Department of Civil Engineering and Energy Technology, OsloMet, Oslo, Norway; 3 Department of Life Sciences and Health, OsloMet, Oslo, Norway

**Keywords:** Kindergarten, Indoor microbiome, Dust analysis, Antibiotic resistance, Built environment

## Abstract

**Background:**

In Norway, 91% of children aged 1–5 attend kindergarten where they are exposed to indoor microbiomes which can have relevance for development and health. In order to gain a better understanding of the composition of the indoor microbiome and how it is affected by occupancy over time, floor dust samples from a newly opened kindergarten were investigated. Samples were collected during an 11-month period. Samples were analyzed for bacterial composition using 16S rRNA gene sequencing. Samples were also screened for four clinically relevant antibiotic resistance genes. In addition, Petrifilm analyses were used to evaluate surface hygiene.

**Results:**

Significant changes in the microbial community composition were observed over time (PERMANOVA, *P* < 0.05). Particularly, changes in the abundance and the proportions of human associated bacteria were found. A decrease in the prevalence of *Propionibacterium* from over 16% abundance to less than 1% and an increase in *Streptococcus* from 10 to 16% were the most significant findings. Four classes of clinically relevant antibiotic resistance genes were tested for; three were detected in the dust, indicating the presence of resistant bacteria and a potential for resistance spread. Petrifilm analysis showed that some surfaces in the kindergarten were of consistent poor hygienic quality, and new hygienic routines are required.

**Conclusions:**

This study, which is the first of its kind performed at a newly opened kindergarten, reveals changes in the microbiome over time as well as the presence of antibiotic resistance genes and hygiene issues which are of relevance for occupant health.

**Electronic supplementary material:**

The online version of this article (10.1186/s40168-018-0553-x) contains supplementary material, which is available to authorized users.

## Background

In recent years, increased attention has been given to the investigation of the microbial ecology and diversity of our surrounding environments, including the buildings that we inhabit. There is a growing interest concerning the possible effects of microbiomes in the built environment on human microbiomes. Recent advances in DNA-based molecular analyses, coupled with developments in computational software and bioinformatics, have made possible the study of complex microbial communities and the interactions between microorganisms and their environments. Historically, culture-based methods have been used for identification of microorganisms. However, culture-based methods have significant limitations in that less than 1% of environmental microorganisms are readily detectable using the culture-based approach [1, 2]. High-throughput DNA sequencing methods yield datasets that provide more information about microbial communities than was possible to generate in earlier classical studies.

With the advent of high-throughput DNA sequencing, we are beginning to understand how the indoor microbiome is affected by the people who inhabit the buildings and by technical installations, design choices, and geographic location [3, 4]. It has been shown that different buildings and different room types can display unique microbiomes [5, 6]. Many of the studies to date have been conducted in buildings such as private housing, offices, schools, and hospitals [6–9]. Relatively few studies have investigated the microbiome of kindergartens [10–12]. Some investigations have documented how microorganisms come to inhabit buildings, both after construction and after families have moved to new houses [6, 7]. In addition, relatively little is known about how the built microbiome can affect human health [13–15]. Given that humans spend about 90% of their time indoors, it is likely that the indoor climate has a significant effect on our health and development.

Kindergartens are an indoor environment where many small children spend a significant amount of time throughout the day. In Norway, 91% of children aged 1–5 years attend kindergartens, whereas in the age range 3–5 years, as many as 97% attend them. In addition, 95% of the children who attend kindergarten in Norway spend more than 41 h per week there [16]. It has been shown that kindergarten attendance can lead to an increased risk for diseases such as otitis media [17] and respiratory illness [18, 19].

It is widely suspected that the human microbiota play a role in the development of several disorders, such as diabetes, asthma, allergies, and obesity. The development of the gut microbiota in children has also been found to play an important part in the maturation of the immune system [20]. Humans attain their microorganisms primarily after birth. The uterine environment was long considered to be sterile. However, we now know that there is exposure to some microorganisms in utero [21]. The bacteria in the human microbiota are also acquired through exposure to our surroundings after birth, and this eventually leads to the development of microbial communities that are unique to each person. Landmark events from birth through infancy and childhood play pivotal roles in the shaping of our microbiota [22–24]. When children reach the age of 3–5, the gut microbiota is starting to resemble that of an adult in terms of its composition and diversity, and microbiomes transition from dynamic to more static ones [23, 25].

Large numbers of airborne bacteria indoors have been found to arise from resuspension of floor dust [26]. Exposure to floor dust might potentially have a larger impact on children than adults as the former are in a more active developmental phase, particularly with regard to immune function. One study suggests that concentrations of resuspended floor dust bacteria can be as much as 9–21 times higher close to the floor in the infant breathing zone than further up in the adult breathing zone [27, 28]. Characterization of floor dust might then provide useful information on exposure to environmental bacteria, especially when considering environments primarily inhabited by small children.

Antibiotic resistant bacteria (ARB) are a major and growing global health concern. Antibiotic resistance is conferred through mutation events and through the acquisition of antibiotic resistance genes (ARG). ARG are often found in the human microbiota, and some studies have shown that ARG can be found in the gut microbiota of young infants, even if they have not been exposed to antibiotics [29]. As humans emit large numbers of bacteria to their surroundings, it is likely that bacteria carrying ARG can be found in the built microbiome. In one study, resistant strains of airborne culturable *Staphylococcus aureus* were present in higher concentrations inside the study homes than outside the homes [30]. However, only limited research has been done on the transmission of antibiotic resistance and ARB in our domestic and work environments. Furthermore, we still know little about how the indoor environment can function as a reservoir for ARG.

In the present study, we investigated, with particular emphasis on floor dust, the bacterial diversity and the presence of ARG in three different rooms in a recently constructed kindergarten in Oslo, Norway. Previous studies have shown that in other indoor environments, including hospitals and housing, human occupancy shapes the structure of indoor microbial communities, which take on the microbial signatures of the occupants [6, 7]. Furthermore, Lax et al. [6] showed that these changes can occur rapidly. The primary aim of this study was to investigate if a similar effect could be seen in kindergartens and if the different rooms acquired different bacterial taxa that could be related to their primary use. In addition, to gain information about the number of viable, readily culturable bacteria, including commonly studied indicator bacteria, surface-hygiene analyses using both general and indicator-selective Petrifilms were included in the study.

## Methods

Sampling was performed five times during the course of 11 months, beginning shortly after the recently constructed kindergarten in Oslo, Norway, was opened. Floor dust samples were analyzed for bacterial composition by 16S rRNA gene amplicon targeted metagenomic analysis. Floor dust samples were also analyzed for the presence of four clinically relevant bacterial ARG using targeted ARG polymerase chain reaction (PCR) primers. In addition, microbial activity on selected surfaces in each of the rooms sampled was investigated using Petrifilm® analyses (3M, MN, USA).

### Sampling

Settled floor dust samples were collected from three separate rooms. The first room was the main activity room (approx. 50 m^2^) which is primarily used for play activities for the children. In connection with the activity room is also a kitchenette area for food preparation. The second room was the toilet (approx. 20 m^2^) which had three stalls and two diaper changing stations along with hand-wash basins. The toilet was located adjacent to, but closed off from the main activity room. The third room was a kitchen located in the opposite end of the building from the main activity room. This room is mainly used by staff. Five rounds of sampling were performed in the period February 2015 to January 2016. The first sampling round took place on February 11, 2015, 3 weeks after the initial opening of the kindergarten. The second sampling round took place on April 23, 2015. The third sampling round took place on August 20, 2015. The fourth sampling round was on December 3, 2015, and the fifth and final sampling round took place on January 5, 2016. All samples from each round were collected and processed on the same day. Dust samples were collected from the floors by vacuuming an area of at least 10 m^2^ of the total floor in each room providing approximately 500 mg of dust for DNA extraction. Sampling was performed using a Flite2 area sampling pump equipped with a sterile plastic 0.4-μm polycarbonate micro-vacuum cassette (SKC Inc., PA, USA). Blank controls were taken by collecting and extracting heat-sterilized DNA-free glass wool from sterile microvacuum cassettes. Blank control samples were then processed and sequenced using the same protocols as the collected dust samples.

### DNA extraction

DNA was extracted from 60 to 100 mg dust using the PowerWater® DNA isolation kit (MO BIO, CA, USA). As previously described [11], dust was weighed into a bead-beater tube (BIOSPEC, OK, USA), and then 1 ml of prewarmed (55 °C) PW1 from the kit was added to the tube and the dust was mixed into the solution using a pipette tip. Tubes were maintained at 55 °C for 10 min. The remainder of the tube was then filled by the addition of beadmix from the kit. The dust/bead mix was homogenized using μ-MiniBeadbeater (BIOSPEC) set at maximum effect for 5 min. The liquid fraction of the tube was transferred to an Eppendorf tube and centrifuged at 13000×*g* for 1 min. The aqueous phase was transferred to a new Eppendorf tube, taking care to avoid the precipitate. Thereafter, 200 μl of PW2 from the kit was added, and the supplied protocol was followed to completion. Final elution of the DNA was with 75 μl of molecular biology grade water prewarmed to 55 °C. Eluted DNA was quantified, and the 260/280 ratio was measured using a nanodrop device (Thermo Fisher Scientific, MA, USA). DNA was stored at − 20 °C until required for downstream applications. Blank controls yielded no measurable levels of DNA.

### 16S rRNA gene analysis

PCR amplification was performed using the universal bacterial 16S rRNA gene primers 27F (5′-AGAGTTTGATCCTGGCTCAG-3′) and 338R (5′-TGCTGCCTCCCGTAGGAGT-3′), which amplify the V1–V2 regions of the 16S rRNA gene [31]. Primers were designed for use with the Roche 454 sequencing platform (454 Life Sciences, Branford, CT, USA). Adaptors and multiplex identifiers (MIDs) were included in primer sequences. PCR reactions were carried out in a total volume of 50 μl with the following components: 10 μl 5× Flexi buffer (Promega, Fitchburg, WI, USA), 2 μl dNTPs (10 mM, Promega), 0.2 μl GoTaq polymerase (5 U/μl, Promega), 3 μl MgCl_2_ (25 mM, Promega), 31.3 μl certified nucleic-acid free water (Invitrogen, Waltham, MA, USA), 1 μl (25 μM) forward primer (MWG Eurofins, Ebersberg, Germany), 1 μl (25 μM) reverse primer (Eurofins), and 1.5 μl template DNA. PCR conditions were as follows: 5 min initial denaturation at 94 °C; 35 cycles of 60 s at 94 °C, 45 s at 55 °C, and 90 s at 72 °C; and 10 min at 72 °C for final extension. PCR amplicons were size controlled and quantified by gel electrophoresis using Low Mass DNA Ladder standards (Invitrogen). Amplicon concentrations were calculated using a Bio Rad Gel Doc XR+ system with Bio Rad Image Lab software (Hercules, CA, USA). Triplicate reactions for each sample were pooled at equimolar concentrations prior to sequencing. Sequencing was performed by Eurofins Genomics laboratories.

### Detection of resistance genes

DNA samples were analyzed for the presence of four ARG-classes: *mecA* (methicillin resistance), *ermA* (macrolide-lincosamide-streptogramin B resistance), *vanA* (glycopeptide resistance), and *aac(6′)-aph(2″)* (aminoglycoside resistance) (Table 1), using gene-specific primers (Table 2). PCR conditions were as described in Table 2. PCR products were detected in agarose gels by staining with Sybr Gold (Molecular Probes, Eugene, OR, USA) to maximize detection sensitivity. All ARG detections included PCR reactions with positive and negative control DNA from clinical bacterial isolates. Table 1 provides an overview of the control strains used for estimating the sensitivity of the detection method. To estimate the detection limit of the PCR-system, genomic DNA from the positive control reference strains was extracted, and 10× serial dilutions were used to determine the lowest detection limit in the experimental setup. Detection of ARG was confirmed by sequencing of the PCR products from samples and positive controls.

**Table 1:** Antibiotic resistance genes

Target gene	Antibiotic resistance	Positive control strain	Negative control strain
*mecA*	Methicillin resistance	*S. aureus* DSM 11729	*S. aureus* DSM 799
*ermA*	Macrolide resistance	*S. aureus* N315 A1-17	*S. aureus* DSM 799
*aac(6′)-aph(2″)*	Aminoglycocide resistance	*E. faecium* K60-39	*E. faecalis* ATCC 29212
*vanA*	Vancomycin resistance	*E. faecium* A1-22	*E. faecalis* ATCC 29212

**Table 2:** PCR primers targeting genes coding for antibiotic resistance

Gene	Primer sequence	PCR conditions	Approximate amplicon size (bp)
*mecA*	5′-GTAGAAATGACTGAACGTCCGATAA-3′ 5′-CCAATTCCACATTGTTTCGGTCTAA-3′ [51]	4 min 94 °C;30 cycles (45 s at 94 °C; 45 s at 50 °C; 60 s at 72 °C); 2 min at 72 °C	310
*ermA*	5′-GTTCAAGAACAATCAATACAGAG-3′ 5′-GGATCAGGAAAAGGACATTTTAC-3′ [52]	5 min 94 °C; 30 cycles (30 s at 94 °C; 30 s at 52 °C, 60 s at 72 °C); 10 min 72 °C	421
*aac(6′)-aph(2″)*	5′-TTGGGAAGATGAAGTTTTTAGA-3′ 5′-CCTTTACTCCAATAATTTGGCT-3′ [53]	3 min 95 °C; 35 cycles (20 s at 95 °C; 20 s at 57 °C; 30 s at 72 °C); 2 min 72 °C	174
*vanA*	5′-CATGAATAGAATAAAAGTTGCAATA-3′ 5′-CCCCTTTAACGCTAATACGATCAA-3′ [54]	10 min 95 °C; 30 cycles (30 s at 94 °C; 30 s at 58 °C; 30 s at 72 °C); 10 min 72 °C	1030

### Petrifilm® analysis

Samples were collected from 12 selected surfaces (Table 3). Samples were analyzed using general purpose Aerobic Count (AC) and selective petrifilms (*Enterobacteriaceae* Count (ENT), Staph Express Count (STX), Yeast and Mold Count (Y&M)). The general criteria for choice of sampling points were (a) multiple separated points in each room, (b) frequency of use (e.g., eating table, play area), (c) suspected relevance for health, and (d) practicality (surfaces such as carpets cannot be easily analyzed using Petrifilm). Petrifilms were prepared according to the 3M Environmental Monitoring Procedures manual [32]. In brief, films were hydrated with 1 ml sterile water and stored for hydration as indicated. Surface sampling was done in accordance with the direct contact sampling procedure [32]. Sampling was performed at each site in either duplicate or triplicate. After sampling, the films were transported to our laboratories and incubated as directed in the manual prior to colony counting. Colony counts were classified according to 3M Petrifilm interpretation guides and reported as colony forming units (CFU). CFU counts on ENT, STX, and Y&M were counted manually. CFU counts on AC plates were counted electronically using a 3M Petrifilm plate reader.

**Table 3:** Petrifilm surface sampling sites

Sampling site	Room	Surface description
A	Main	Window ledge
B	Main	Square activity table
C	Main	Round activity table
D	Main	Countertop surrounding sink in kitchenette area
E	Toilet	Tiled wall above basin between diaper changing stations
F	Toilet	Tiled wall outside toilet cubicle
G	Toilet	Tiled wall above children’s hand washing basin
H	Kitchen	Wall above trash cans
I	Kitchen	Countertop surrounding sink
J	Kitchen	Ventilation hood above kitchen stove

### 16S rRNA gene amplicon analysis

Sequences were processed using the default parameters in QIIME version 1.9.1. [33]. Sequences were demultiplexed, and adapters were trimmed. Sequences were removed if they were less than 200 basepairs in length, had a quality score below 25, contained more than six ambiguous bases, or had primer mismatches. After quality control, the remaining high-quality sequences were assigned into operational taxonomic units (OTUs) using the open reference OTU picking strategy at a 97% sequence similarity cutoff. Representative sequences were then aligned against the Greengenes 16S rRNA gene database v13.8 [34]. Mitochondrial and chloroplast sequences were removed prior to downstream analysis. To eliminate potential bias due to sampling depth, all samples were then rarefied to a sequencing depth lower than the smallest sequencing depth (1600) prior to alpha and beta diversity analysis. To control for sample processing contamination, sequences generated from blank controls were plotted along with the samples in principal coordinate analysis (PCoA) plots to ensure that no samples were clustered close to the negative controls (Additional file 1: Figure S1) [27].

### Statistical analysis

Data analysis and visualization was performed with R, primarily using functions from R package phyloseq, vegan, and ggplot2 [35]. For beta diversity community analysis, Bray-Curtis distances were used to produce PCoA plots to compare phylogenetic distances between samples. Permutational analysis of variance (PERMANOVA) employing ADONIS in R (Package: vegan) was used to compare Bray-Curtis distances against sampling round or room type. The number of permutations was set at the default 999 to calculate *P* values. To look for differences in alpha diversity across room types or sampling dates, the Kruskal-Wallis test was applied.

The 3M plate counter accurately counts colonies up to 999 colonies per plate. Counts above 999 colonies are reported as too numerous to count (TNTC). For statistical analysis, plates reported as TNTC were given a CFU count of 1000, i.e., just above the upper range of accurate counting.

## Results and discussion

### Taxonomic analysis

After sequence filtering and quality control of 15 samples taken from 5 sampling rounds in three rooms, a total of 355,293 sequence reads, with a median/average of 23,838/23,686 sequence reads per sample (min 4928, max 42,382) were produced. This corresponded to 3678 taxa. After chloroplast and mitochondrial sequences were removed, all samples were rarefied to a sequencing depth of 1600 sequences per sample. After rarefaction, 2120 unique taxa were obtained across all samples.

An average of 391 taxa was observed in all the samples (min 223, max 534). The average Chao1 index estimate for all the samples was 694.7 (min 283.4, max 891.5). There were no significant differences in the numbers of observed taxa in samples across sampling rounds (Kruskal-Wallis test, df = 4, *P* > 0.05) or room types (Kruskal-Wallis test, df = 2, *P* > 0.05). Furthermore, no significant differences were observed in Chao1 estimates when samples were categorized by either sampling round (Kruskal-Wallis test, df = 4, *P* > 0.05) or room type (Kruskal-Wallis test, df = 2, *P* < 0.05) (Additional file 1: Table S1).

#### Effect of sampling round and room type on microbiome composition

**Fig. 1:**
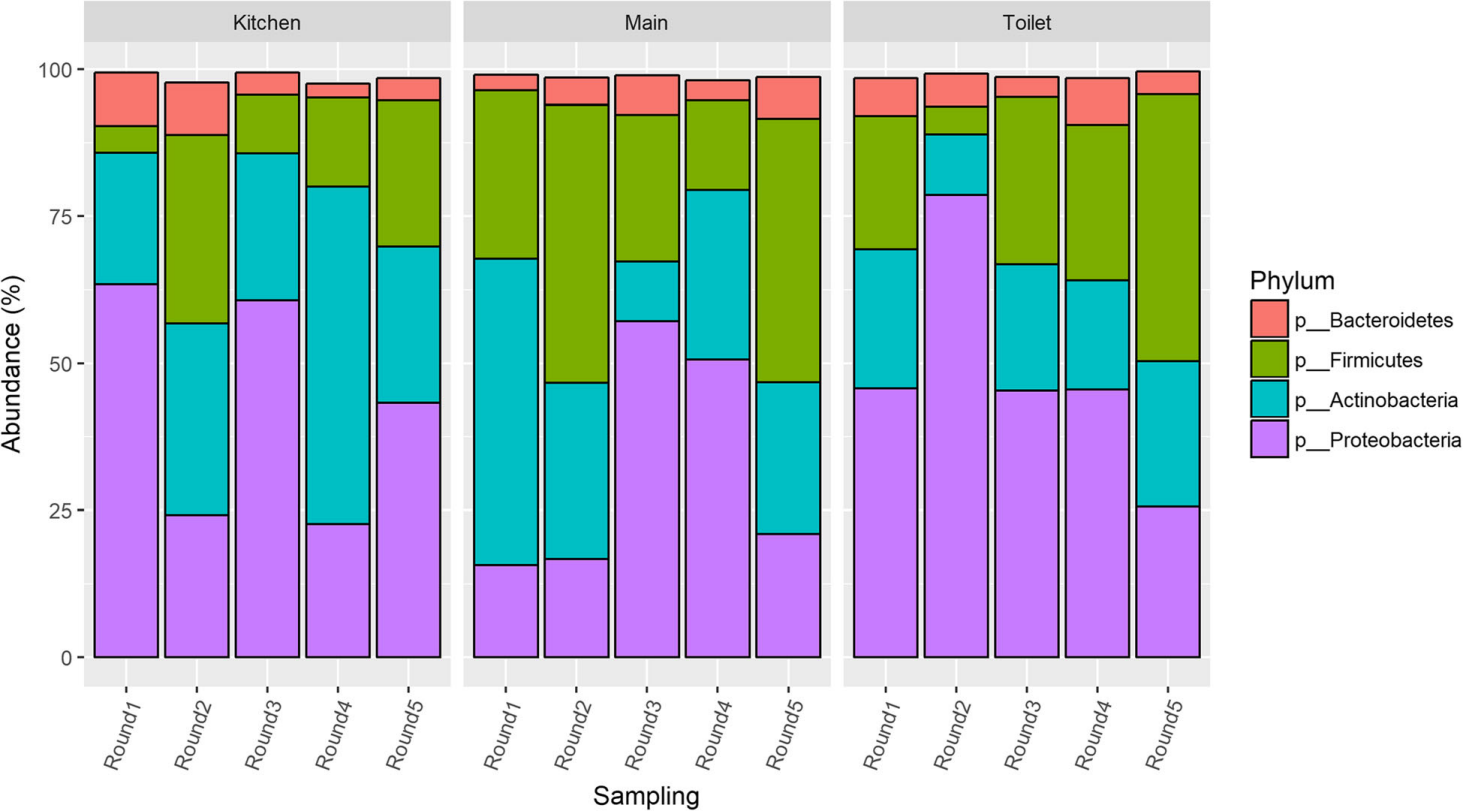
Changes in bacterial composition at phylum level over time in the kindergarten

At the phylum level, analysis of the taxonomic composition showed that more than 98% of the sequences were classified as *Proteobacteria*, *Firmicutes*, *Actinobacteria*, and *Bacteroidetes*. The most abundant phylum was *Proteobacteria* (41.0% of sequences), followed by *Actinobacteria* (27.3%), *Firmicutes* (25.0%), and *Bacteroidetes* (5.3%) (Fig. 1). These phyla remained dominant throughout the 11-month sampling period. The phyla observed in kindergarten floor dust are also those most commonly found in residential housing as well as schools and other indoor environments. Furthermore, they are also the chief components of the human skin microbiota [36]. The results may indicate that major changes at the phylum level do not occur as a consequence of room habitation and use. Alternatively, phylum level analysis may be too general to reveal significant changes in bacterial communities if these occur. At the genus level, some trends were seen (see below).

When chloroplast sequences were retained in the dataset, it was seen that they made up more than 78.9% of the sequences in the kitchen floor dust samples (Additional file 1: Figure S2). This is of potential interest as the proportion of chloroplast sequences could provide an indication of the fundamental nature and properties of the dust itself. In a study of ventilation filter dust in kindergartens, we found that 35% of the sequences in the intake filter (i.e., outdoor air) were identified as chloroplast DNA, whereas less than 6% of sequences identified from exhaust filters (i.e., indoor air) were from chloroplasts [11]. However, it seems pertinent to remove chloroplast sequences as they are not directly relevant to an investigation of bacterial dispersal in the building.

#### Genus-level composition

**Fig. 2:**
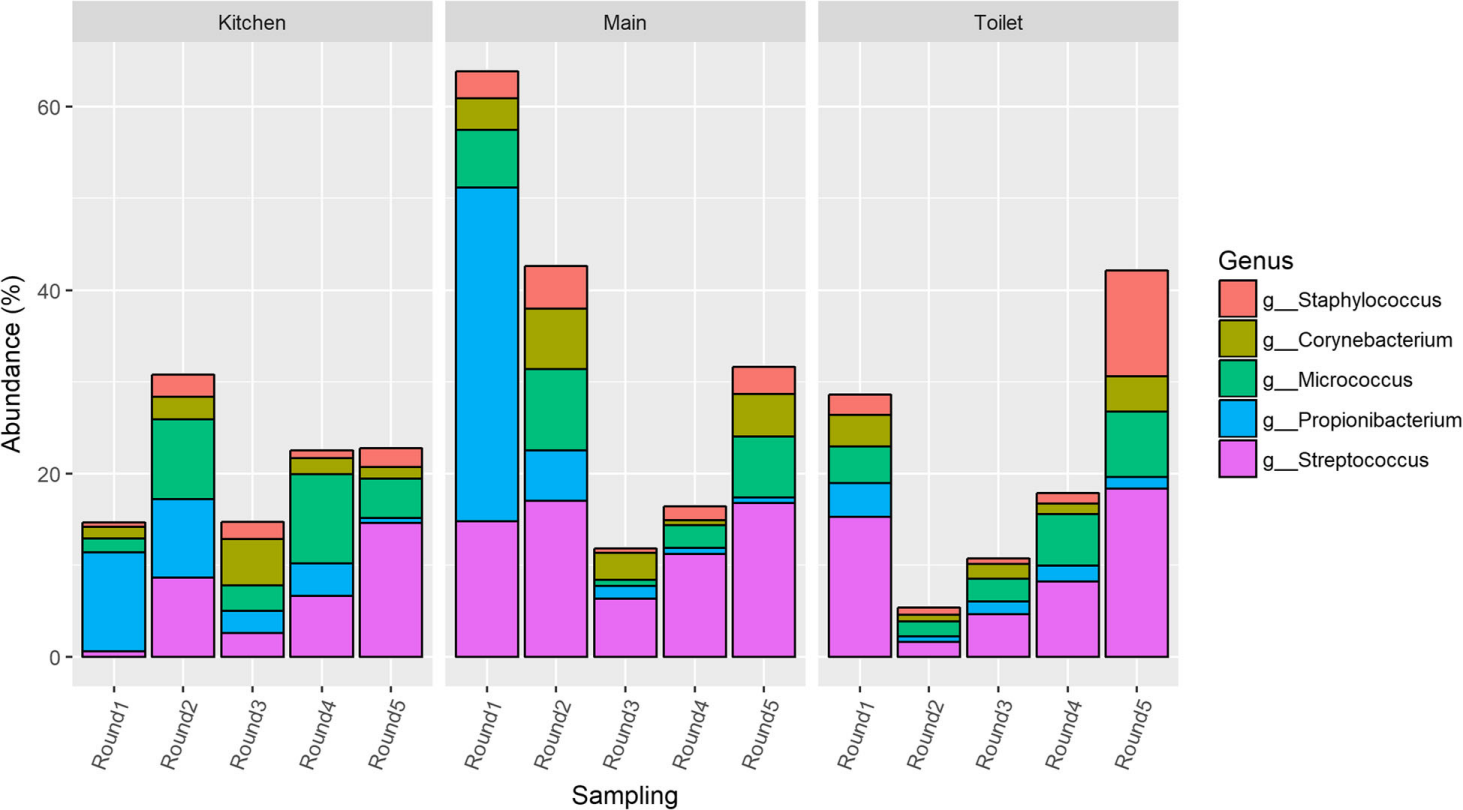
Changes in the relative abundance of selected human-associated related bacteria over time in the kindergarten

Among the most abundant genera were several that have been identified in previously published studies of the indoor microbiome [37, 38]. Floor dust samples in the present study were generally dominated by genera such as *Janthinobacterium*, *Micrococcus*, *Staphylococcus*, *Streptococcus*, *Corynebacterium*, *Propionibacterium*, and *Microbacterium*. To highlight the possible impact of human activity on the bacterial composition of the floor dust, data on selected human-associated genera (*Micrococcus*, *Staphylococcus*, *Streptococcus*, *Corynebacterium*, *Propionibacterium*) and their relative abundances were extracted and are presented in Fig. 2. On average, human-associated genera made up 25% of the relative abundance across all samples. In the first sampling in the main room, they made up more than 60% of the relative abundance, largely due to a high abundance of *Propionibacterium*.

For the main room and the kitchen, the abundance of human-associated bacteria was highest in the early and late sampling rounds (Fig. 2). This could possibly be explained by seasonal variation, as both the first and fifth sampling were performed during winter months (December and January, respectively), when staff and children spend more time indoors. The use of natural ventilation (opening of windows) might also be lower during winter months. Studies have shown seasonal variation in indoor bacterial exposures. Frankel et al. [39] showed that indoor CFU concentrations for bacteria were highest during spring and fall and lowest during summer. In the present study, the relative abundance of *Propionibacterium* decreased over time in all rooms (Fig. 2). It is possible that a source of *Propionibacterium* was present in the building only during the earlier sampling rounds. *Propionibacterium* is one of the main commensals of the skin microbiome and is mainly found on sebaceous (oily) skin sites in adolescents and adults [40, 41]. As sebaceous glands mature during puberty, the onset of puberty is often accompanied by an increase of *Propionibacterium* in the skin microbiome. Initially, the building was solely occupied by adults, i.e., those involved in the construction process. After construction, the primary occupants of the building were pre-pubescent children. This could potentially explain the decrease in *Propionibacterium* over time, and its comparatively low abundance in kindergartens compared to other indoor environments. Shin et al. [10] looking at bioaerosols in childcare facilities found *Propionibacterium* to have the lowest abundance of the human-associated bacteria. Interestingly, other studies of the indoor dust microbiome have found the relative abundance of *Propionibacterium* to be generally higher than in the present work [26, 42]. A study by Hospodsky et al. [26] looking at indoor microbiomes in university classrooms found *Propionibacteriaceae* to be consistently one of the five most abundant taxa in floor dust, HVAC filter dust, indoor air, and ventilation supply air. Further work could investigate the role of building occupant age on the abundance of *Propionibacterium* in the indoor environment.

The last three rounds of sampling (four for the toilet) showed a gradual increase in the abundance of *Streptococci*. This might be explained by gradual colonization of the building in this period. However, as a high abundance of *Streptococci* was also found in some of the earlier samples, caution is required in the interpretation of the data. An identical trend was seen for the *Micrococci* in the main room and toilet. As *Streptococci* are associated with a number of illnesses with special relevance for children (e.g., impetigo), this finding could potentially be important.

#### Phylogenetic composition

Community composition analysis showed that only sampling round was found to be a significant predictor of the bacterial community composition (df = 4, F.model = 1.312, *R*^2^_Adonis_ = 0.340, *P*_Adonis_ = 0.046*). Room type was not found to have a significant impact on phylogenetic dissimilarity (df = 2, F.model = 1.104, *R*^2^_Adonis_ = 0.143, *P*_Adonis_ = 0.256). These results suggest that the bacterial composition in kindergarten dust is more influenced by sampling round, possibly related to accumulation over time or seasonal variation, than by room type. The data in Fig. 2 suggests that there may be higher concentrations of human-associated bacteria in the winter months. However, unlike indoor fungal populations, indoor bacterial populations have been found to be less driven by seasonal variation [43, 44]. A study of seasonal variation in airborne bacteria and viruses in a children’s daycare center did not find clear evidence of seasonal variation in bacterial communities, but did find seasonal patterns for some respiratory viruses [12].

As the kindergarten was newly opened, it was hypothesized that its microbiome might change fundamentally as a consequence of occupancy and perhaps eventually settle to a more stable composition. Changes in the relative abundance of *Propionibacterium* and *Streptococcus* are noted above and provide some support for this. That the appearance of these genera indoors in nurseries which are associated with occupancy is supported by our previous work on ventilation in kindergartens [11]. This study showed that these bacteria were close to absent in intake dust samples but among the most abundant genera in exhaust filter dust. To date, few longitudinal studies have been done to examine how the indoor microbiome develops over time. A longitudinal study of the indoor microbiome by Lax et al. [6] found that when families moved to new dwellings, the indoor microbiome of the dwellings would be rapidly shaped by the family, i.e., the new dwelling became populated by the inhabitants’ microbiome. A similar trend has been observed in the hospital environment, where bacterial communities on patients and room surfaces became increasingly similar over the course of a patient’s stay [7].

### Antibiotic resistance gene detection

Four ARG (Table 1) were screened for using PCR in 15 floor dust samples. Table 4 shows the results from the ARG PCR assay. The genes *mecA*, *ermA*, and *aac(6′)-aph(2″)* were detected in all samples, whereas the *vanA* gene was not detected in any of the samples. The lower limit of PCR detection for the four ARG classes based on the results for the positive control strains is shown in Table 4.

**Table 4:** Antibiotic resistance genes detected in floor dust samples

Resistance gene	Primary host species	Presence (+)/absence (−) of gene			Detection limit range (pg)
		Main	Toilet	Kitchen	
*mecA*	*Staphylococcus*	+	+	+	2181.7–218.87
*ermA*	*Staphylococcus*	+	+	+	1.206–0.121
*aac(6′)-aph(2″)*	*Staphylococcus/Enterococcus*	+	+	+	9.35–0.935
*vanA*	*Staphylococcus/Enterococcus*	–	–	–	261.1–26.11

In a study of vacuum cleaner dust and air samples at an Australian university, no samples tested positive for the presence of *ermA* or *vanA*, whereas a number of other resistance genes were found by endpoint PCR. *MecA* and *aac(6′)-aph(2″)* were not tested for [45]. In contrast, analysis of ventilation filter dust in a hospital showed the presence of *ermA*, *mecA*, and *aac(6′)-aph(2″)*, whereas *vanA* was not detected [46]. These results are similar to those for kindergarten dust tested in the present study. Drudge et al. concluded that the presence of *mecA* together with *aac(6′)-aph(2″)* and *ermA* could be due to multidrug-resistant *methicillin resistant Staphylococcus aureus* (MRSA). It is perhaps unlikely that a newly opened kindergarten should house multidrug-resistant MRSA. However, if the suggestion made by Drudge et al. is likely to be true, then, dust samples from kindergartens should be further analyzed for MRSA. It is important to recognize that *mecA* can also be carried by coagulase-negative strains of *Staphylococcus*, such as *Staphylococcus epidermidis*. However, according to Drudge et al. [46], the finding of *mecA*, *aac(6′)-aph(2″)*, and *ermA* is perhaps some cause for concern, as these genes can be found on mobile genetic elements [47, 48]. Further studies could look at the resistance properties of putative *S. aureus* obtained on STX petrifilm.

The abundance of ARG in environmental samples remains largely unknown. PCR-based methods, such as those used in the present study, can provide insight into the commonality of such genes in our surroundings. Although these methods do not distinguish between viable and nonviable bacteria, they provide knowledge concerning the abundance and spread of ARG and help us to understand how antibiotic resistance capacities can potentially spread through the indoor environment.

Shotgun sequencing-based metagenomic studies could provide more comprehensive information concerning a wider range of ARG. Notwithstanding, the endpoint PCR-based method provides a simple procedure for detecting ARG in dust and other environmental samples.

### Petrifilm analysis

Petrifilm is a much used technique in the food industry which can also be used for assessing hygienic quality of surfaces [49]. In addition to aerobic counts, *Enterobacteriaceae*, putative *S. aureus*, and yeast and mold were also found in the kindergarten (Table 5).

**Table 5:** Petrifilm analyses summary data

Petrifilm	Mean	Median	SD	Max	Min
Aerobic Plate Count (AC)	320	160	340	1000	0
*Enterobacteriaceae* Count (ENT)	39	0	110	500	0
Staph Express Count (STX)	6	1	10	43	0
Yeast and Mold Count (Y&M)	17	15	14	62	0

**Fig. 3:**
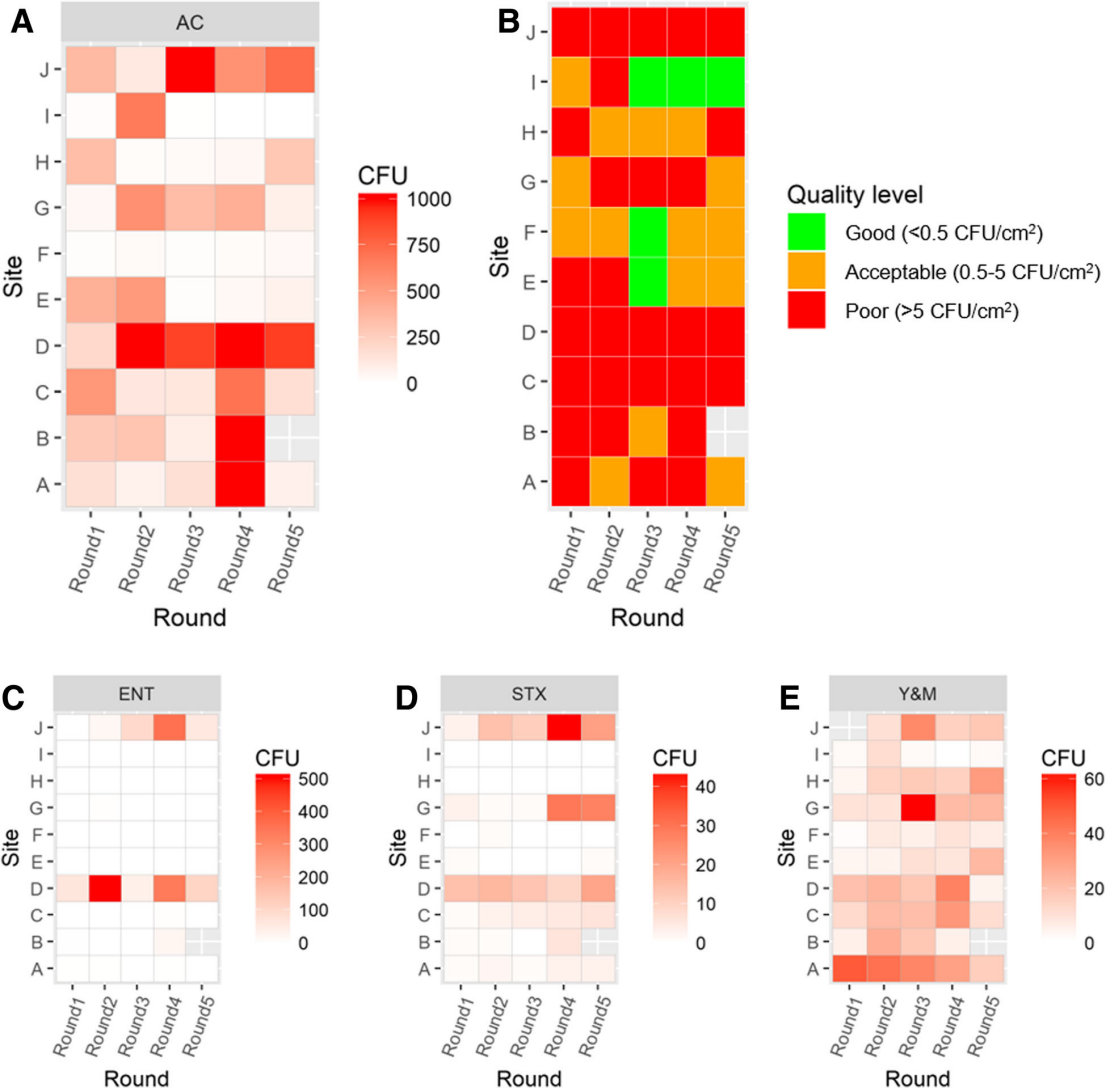
Heatmaps showing Petrifilm counts for **a** aerobic counts, **c** Enterobacteriaceae, **d** Staphylococcus, and **e** yeast and molds. In **b**, aerobic CFU counts are expressed as surface hygiene quality

*Enterobacteriaceae* were primarily found at site D (kitchenette countertop) and J (ventilation hood) (Fig. 3c). Putative *S. aureus* was also primarily found at sites D and J. However, site G (above hand washing basin) also showed high STX counts in the two last rounds of sampling (Fig. 3d). Yeasts and molds were found to be more ubiquitous throughout the building, and Y&M counts showed little site variation (Fig. 3e).

There are few international standards and guidelines for interpreting surface hygiene with respect to relevance for health. However, several national food safety agencies have recommended hygienic guidelines, and these have been applied in the food industry. In these instances, it has been suggested that microbial counts should not exceed 5 CFU/cm^2^ [50]. To our knowledge, no guidelines at the present exist for kindergartens. As food is prepared in kindergartens, including the one in the present study, using recommended guidelines for food safety, the observed CFU counts for aerobic bacteria (Fig. 3a) were converted to an expression of surface hygiene (Fig. 3b). Similar guidelines are not available for ENT, STX, and Y&M.

Sites C, D, and J were consistently of poor hygienic quality (Fig. 3b), and this should be addressed by new hygienic routines. Both C and D were surfaces that are likely to have high rates of contact with children and staff. Site J was a ventilation hood which was visibly dirty. This is a surface often omitted during cleaning. However, the findings indicate that it is important to clean this surface, as bacteria could possibly fall down onto the countertop below which is used for food preparation. Site F was the only surface that consistently had acceptable surface quality. Sites D, G, and J are in close vicinity to sources of dampness, i.e., wash basins and cooking stoves. Site I, just below site J, was cleaned regularly and appeared much cleaner compared to J and also showed a higher hygienic level across all plate types.

No *Enterobacteriaceae* was found on surfaces in the toilet area, which could suggest that formation of biofilms containing these bacteria has not occurred. However, *Enterobacteriaceae* was present in the buildings and was found consistently at sites D and J, both of which are in close vicinity to food preparation areas.

## Limitations

The major limitation of this study is that the results are based on only a single kindergarten. Furthermore, it would be desirable to have more than five sampling rounds, in order to better assess temporal variability in the data. Unfortunately, we were unable to collect samples prior to the opening of the kindergarten. This would have been the ideal baseline sample. Notwithstanding, the first sample was collected shortly after opening. In addition, the present study focuses on floor dust. However, as our previous work has shown, HVAC samples could have been included as these can also reveal microbial changes in the indoor space. The strength of the present work is the combination of both culture- and nonculture-based analyses. In addition, few other studies have followed the development of bacterial populations in multiple indoor spaces over such a long period. The inclusion of ARG screening also gives a partial characterization of the kindergarten environment as a reservoir for ARGs. However, future studies based on shotgun sequencing would give a more comprehensive understanding the indoor resistome.

## Conclusion

We examined the bacterial communities of floor dust in a newly opened Norwegian kindergarten. Significant changes in the bacterial community composition could be observed over time, particularly with respect to the abundance and the proportions of human-associated bacteria. Further studies could look to see if the trends observed here with *Propionibacterium* and *Streptococci* are repeated in other kindergarten and daycare settings. We also detected the presence of three clinically relevant ARG in the dust, which could be synonymous with resistant *S. aureus*. Methods such as shotgun metagenomic sequencing should be employed to investigate whether strains of MRSA are present in the dust. This study also shows that Petrifilm can conveniently identify areas of poor hygienic quality. More kindergartens should be examined using similar approaches as used here, to see if similar trends occur. Knowledge of the indoor environment in kindergarten and day care settings holds great potential for understanding how microorganisms in the indoor environments can affect health, as small children might experience more long-lasting health effects of exposure to microorganisms than adults. Our study also indicates the importance of investigating the prevalence of ARB in these environments. Such knowledge may help us understand better the mechanics of community-acquired antibiotic resistant infections and the epidemiological spread of ARB.

## Additional files

Additional file 1: **Table S1.** Alpha diversity measures. **Table S2.** QIIME mapping file for Sample 1 (sample accession number SAMEA4670724). **Table S3.** QIIME mapping file for Sample 2 (sample accession number SAMEA4670725). **Table S4.** QIIME mapping file for Sample 3 (sample accession number SAMEA4670726). **Table S5.** QIIME mapping file for Sample 4 (sample accession number SAMEA4670727). **Table S6.** QIIME mapping file for Sample 5 (sample accession number SAMEA4670728). **Figure S1.** Principal Coordinate plot (PCoA) of Bray-Curtis distances plotting samples and negative controls. **Figure S2.** Changes in the bacterial composition at class level over time in the kindergarten, with chloroplast sequences retained. (DOCX 131 kb)

## References

[1] Staley JT, Konopka A (1985). Measurement of in situ activities of nonphotosynthetic microorganisms in aquatic and terrestrial habitats. Annu Rev Microbiol.

[2] Hugenholtz P (2002). Exploring prokaryotic diversity in the genomic era. Genome Biol.

[3] Prussin AJ, Marr LC (2015). Sources of airborne microorganisms in the built environment. Microbiome.

[4] Adams Rachel I., Bhangar Seema, Dannemiller Karen C., Eisen Jonathan A., Fierer Noah, Gilbert Jack A., Green Jessica L., Marr Linsey C., Miller Shelly L., Siegel Jeffrey A., Stephens Brent, Waring Michael S., Bibby Kyle (2016). Ten questions concerning the microbiomes of buildings. Building and Environment.

[5] Kembel Steven W., Meadow James F., O’Connor Timothy K., Mhuireach Gwynne, Northcutt Dale, Kline Jeff, Moriyama Maxwell, Brown G. Z., Bohannan Brendan J. M., Green Jessica L. (2014). Architectural Design Drives the Biogeography of Indoor Bacterial Communities. PLoS ONE.

[6] Lax S, Smith DP, Hampton-Marcell J, Owens SM, Handley KM, Scott NM, Gibbons SM (2014). Longitudinal analysis of microbial interaction between humans and the indoor environment. Science.

[7] Lax S, Sangwan N, Smith D, Larsen P, Handley KM, Richardson M, Guyton K (2017). Bacterial colonization and succession in a newly opened hospital. Sci Transl Med.

[8] Hewitt KM, Gerba CP, Maxwell SL, Kelley ST (2012). Office space bacterial abundance and diversity in three metropolitan areas. PLoS One.

[9] Meadow JF, Altrichter AE, Kembel SW, Kline J, Mhuireach G, Moriyama M, Northcutt D (2014). Indoor airborne bacterial communities are influenced by ventilation, occupancy, and outdoor air source. Indoor Air.

[10] Shin S-K, Kim J, Ha S-m, Oh H-S, Chun J, Sohn J, Yi H (2015). Metagenomic insights into the bioaerosols in the indoor and outdoor environments of childcare facilities. PLoS One.

[11] Nygaard AB, Charnock C. The bacterial composition of ventilation filter dust in Norwegian pre-school nurseries. Indoor Built Environ. 2017. 10.1177/1420326X17713831.

[12] Prussin AJ, Vikram A, Bibby KJ, Marr LC (2016). Seasonal dynamics of the airborne bacterial community and selected viruses in a children’s daycare center. PLoS One.

[13] Lax S, Nagler CR, Gilbert JA (2015). Our interface with the built environment: immunity and the indoor microbiota. Trends Immunol.

[14] Hoisington AJ, Brenner LA, Kinney KA, Postolache TT, Lowry CA (2015). The microbiome of the built environment and mental health. Microbiome.

[15] Stamper C.E., Hoisington A.J., Gomez O.M., Halweg-Edwards A.L., Smith D.G., Bates K.L., Kinney K.A., Postolache T.T., Brenner L.A., Rook G.A.W., Lowry C.A. (2016). The Microbiome of the Built Environment and Human Behavior. International Review of Neurobiology.

[16] Statistics Norway. Kindergartens, 2017, Final figures. 2017. https://www.ssb.no/en/utdanning/statistikker/barnehager/aar-endelige/2017-03-21. Accessed 10/08/2017

[17] Uhari M, Mäntysaari K, Niemelä M (1996). Meta-analytic review of the risk factors for acute otitis media. Clin Infect Dis.

[18] Marbury MC, Maldonado G, Waller L (1997). Lower respiratory illness, recurrent wheezing, and day care attendance. Am J Respir Crit Care Med.

[19] Hurwitz ES, Gunn WJ, Pinsky PF, Schonberger LB (1991). Risk of respiratory illness associated with day-care attendance: a nationwide study. Pediatrics.

[20] West CE, Jenmalm M, Prescott S (2015). The gut microbiota and its role in the development of allergic disease: a wider perspective. Clin Exp Allergy.

[21] Aagaard K., Ma J., Antony K. M., Ganu R., Petrosino J., Versalovic J. (2014). The Placenta Harbors a Unique Microbiome. Science Translational Medicine.

[22] Bäckhed F, Roswall J, Peng Y, Feng Q, Jia H, Kovatcheva-Datchary P, Li Y (2015). Dynamics and stabilization of the human gut microbiome during the first year of life. Cell Host Microbe.

[23] Greenhalgh K, Meyer KM, Aagaard KM, Wilmes P (2016). The human gut microbiome in health: establishment and resilience of microbiota over a lifetime. Environ Microbiol.

[24] Thompson AL, Monteagudo-Mera A, Cadenas MB, Lampl ML, Azcarate-Peril M (2015). Milk-and solid-feeding practices and daycare attendance are associated with differences in bacterial diversity, predominant communities, and metabolic and immune function of the infant gut microbiome. Front Cell Infect Microbiol.

[25] Rodríguez JM, Murphy K, Stanton C, Ross RP, Kober OI, Juge N, Avershina E, et al. The composition of the gut microbiota throughout life, with an emphasis on early life. Microb Ecol Health Dis. 2015. 10.3402/mehd.v26.26050. 10.3402/mehd.v26.26050 PMC4315782 25651996

[26] Hospodsky D, Qian J, Nazaroff WW, Yamamoto N, Bibby K, Rismani-Yazdi H, Peccia J (2012). Human occupancy as a source of indoor airborne bacteria. PLoS One.

[27] Hyytiäinen HK, Jayaprakash B, Kirjavainen PV, Saari SE, Holopainen R, Keskinen J, Hämeri K (2018). Microbiome.

[28] Wu T, Täubel M, Holopainen R, Viitanen A-K, Vainiotalo S, Tuomi T, Keskinen J (2017). Infant and adult inhalation exposure to resuspended biological particulate matter. Environ Sci Technol.

[29] Gibson MK, Crofts TS, Dantas G (2015). Antibiotics and the developing infant gut microbiota and resistome. Curr Opin Microbiol.

[30] Gandara A, Mota LC, Flores C, Perez HR, Green CF, Gibbs SG (2006). Isolation of Staphylococcus aureus and antibiotic-resistant Staphylococcus aureus from residential indoor bioaerosols. Environ Health Perspect.

[31] Flores GE, Bates ST, Knights D, Lauber CL, Stombaugh J, Knight R, Fierer N (2011). Microbial biogeography of public restroom surfaces. PLoS One.

[32] 3M, Environmental Monitoring Procedures, 3M Food Safety, Editor. 2015, 3M. https://multimedia.3m.com/mws/media/241111O/environmental-monitoring-procedures-article.pdf.

[33] Caporaso JG, Kuczynski J, Stombaugh J, Bittinger K, Bushman FD, Costello EK, Fierer N (2010). QIIME allows analysis of high-throughput community sequencing data. Nat Methods.

[34] DeSantis TZ, Hugenholtz P, Larsen N, Rojas M, Brodie EL, Keller K, Huber T, Dalevi D, Hu P, Andersen GL (2006). Greengenes, a chimera-checked 16S rRNA gene database and workbench compatible with ARB. Appl Environ Microbiol.

[35] Wickham H (2009). ggplot2: elegant graphics for data analysis.

[36] Nakatsuji T, Chiang H-I, Jiang SB, Nagarajan H, Zengler K, Gallo RL (2013). The microbiome extends to subepidermal compartments of normal skin. Nat Commun.

[37] Hoisington A., Maestre J. P., Kinney K. A., Siegel J. A. (2015). Characterizing the bacterial communities in retail stores in the United States. Indoor Air.

[38] Jeon YS, Chun J, Kim BS (2013). Identification of household bacterial community and analysis of species shared with human microbiome. Curr Microbiol.

[39] Frankel Mika, Bekö Gabriel, Timm Michael, Gustavsen Sine, Hansen Erik Wind, Madsen Anne Mette (2012). Seasonal Variations of Indoor Microbial Exposures and Their Relation to Temperature, Relative Humidity, and Air Exchange Rate. Applied and Environmental Microbiology.

[40] Moissl-Eichinger C, Probst AJ, Birarda G, Auerbach A, Koskinen K, Wolf P, Holman H-YN. Human age and skin physiology shape diversity and abundance of Archaea on skin. Sci Rep. 2017. 10.1038/s41598-017-04197-4. 10.1038/s41598-017-04197-4 PMC5481324 28642547

[41] Grice EA, Segre JA (2011). The skin microbiome. Nat Rev Micro.

[42] Qian J, Hospodsky D, Yamamoto N, Nazaroff WW, Peccia J (2012). Size-resolved emission rates of airborne bacteria and fungi in an occupied classroom. Indoor Air.

[43] Adams RI, Miletto M, Lindow SE, Taylor JW, Bruns TD (2014). Airborne bacterial communities in residences: similarities and differences with Fungi. PLoS One.

[44] Adams RI, Miletto M, Taylor JW, Bruns TD (2013). Dispersal in microbes: fungi in indoor air are dominated by outdoor air and show dispersal limitation at short distances. ISME J.

[45] Veillette M, Knibbs LD, Pelletier A, Charlebois R, Lecours PB, He C, Morawska L (2013). Microbial contents of vacuum cleaner bag dust and emitted bioaerosols and their implications for human exposure indoors. Appl Environ Microbiol.

[46] Drudge CN, Krajden S, Summerbell RC, Scott JA (2012). Detection of antibiotic resistance genes associated with methicillin-resistant Staphylococcus aureus (MRSA) and coagulase-negative staphylococci in hospital air filter dust by PCR. Aerobiologia.

[47] Seyedmonir E, Yilmaz F, Icgen B (2015). mecA gene dissemination among staphylococcal and non-staphylococcal isolates shed in surface waters. Bull Environ Contam Toxicol.

[48] Haaber Jakob, Penadés José R., Ingmer Hanne (2017). Transfer of Antibiotic Resistance in Staphylococcus aureus. Trends in Microbiology.

[49] Claro T, O'reilly M, Daniels S, Humphreys H (2015). Surface microbial contamination in hospitals: a pilot study on methods of sampling and the use of proposed microbiologic standards. Am J Infect Control.

[50] Dancer SJ (2004). How do we assess hospital cleaning? A proposal for microbiological standards for surface hygiene in hospitals. J Hosp Infect.

[51] Geha DJ, Uhl JR, Gustaferro CA, Persing DH (1994). Multiplex PCR for identification of methicillin-resistant staphylococci in the clinical laboratory. J Clin Microbiol.

[52] Lina G, Quaglia A, Reverdy M-E, Leclercq R, Vandenesch F, Etienne J (1999). Distribution of genes encoding resistance to macrolides, lincosamides, and streptogramins among staphylococci. Antimicrob Agents Chemother.

[53] Martineau F, Picard FJ, Grenier L, Roy PH, Ouellette M, Bergeron MG (2000). Multiplex PCR assays for the detection of clinically relevant antibiotic resistance genes in staphylococci isolated from patients infected after cardiac surgery. J Antimicrob Chemother.

[54] Clark N, Cooksey R, Hill B, Swenson J, Tenover F (1993). Characterization of glycopeptide-resistant enterococci from US hospitals. Antimicrob Agents Chemother.

