# Supplementary material for: Longitudinal development of the dust microbiome in a newly opened Norwegian kindergarten
Source: Microbiome. 2018 Sep 15;6:159. doi: 10.1186/s40168-018-0553-x (PMC6138906; doi:10.1186/s40168-018-0553-x)
Supplement: Supplementary file 1 — Table S1. Alpha diversity measures. Table S2. QIIME mapping file for Sample 1 (sample accession number SAMEA4670724). Table S3. QIIME mapping file for Sample 2 (sample accession number SAMEA4670725). Table S4. QIIME mapping file for Sample 3 (sample accession number SAMEA4670726). Table S5. QIIME mapping file for Sample 4 (sample accession number SAMEA4670727). Table S6. QIIME mapping file for Sample 5 (sample accession number SAMEA4670728). Figure S1. Principal Coordinate plot (PCoA) of Bray-Curtis distances plotting samples and negative controls. Figure S2. Changes in the bacterial composition at class level over time in the kindergarten, with chloroplast sequences retained. (DOCX 131 kb) [file 40168_2018_553_MOESM1_ESM.docx]

**Supplementary information for:**

**Longitudinal development of the dust microbiome in a newly opened Norwegian kindergarten**

Anders B Nygaard^1,2*^ and Colin Charnock^1,3^

^1^Disease and Environmental Exposures research group, Department of Life Sciences and Health, OsloMet – Oslo Metropolitan University, Oslo, Norway

^2^Department of Civil Engineering and Energy Technology, OsloMet, Oslo, Norway

^3^Department of Life Sciences and Health, OsloMet, Oslo, Norway
*Corresponding author

Author emails:

ABN: [anders.b.nygaard@gmail.com](mailto:anders.b.nygaard@gmail.com)

CC: [colin.charnock@oslomet.no](mailto:colin.charnock@oslomet.no)

Institutional address:

OsloMet – Oslo Metropolitan University
PO box 4 St. Olavs plass
NO-0130 Oslo

Norway

Table S1. Alpha diversity measures

| **Room Type** | **Sampling round** | **Observed OTUs** | **Chao1** | **se.chao1** | **Shannon** | **Fisher** |
| --- | --- | --- | --- | --- | --- | --- |
| Kitchen | Round1 | 223 | 283.3818 | 17.478821 | 3.4788563 | 70.43151 |
| Kitchen | Round2 | 506 | 874.8454 | 61.604088 | 5.3480177 | 254.9857 |
| Kitchen | Round3 | 297 | 371.0769 | 18.672424 | 4.370349 | 107.3528 |
| Kitchen | Round4 | 365 | 657.0685 | 55.686286 | 4.0867417 | 147.7335 |
| Kitchen | Round5 | 381 | 546.0123 | 34.135187 | 5.0079846 | 158.2167 |
| Main | Round1 | 358 | 649 | 57.616823 | 4.0827456 | 143.2693 |
| Main | Round2 | 440 | 817.3684 | 67.895702 | 5.0367363 | 200.4245 |
| Main | Round3 | 341 | 866.0732 | 111.41287 | 4.4429533 | 132.7291 |
| Main | Round4 | 458 | 848.1807 | 67.821603 | 5.2575718 | 214.4902 |
| Main | Round5 | 435 | 728.9241 | 54.597591 | 5.1817322 | 196.62 |
| Toilet | Round1 | 497 | 959.1282 | 79.702297 | 5.519834 | 247.0466 |
| Toilet | Round2 | 249 | 571.2632 | 75.140364 | 2.8111984 | 82.6177 |
| Toilet | Round3 | 534 | 891.5 | 57.118552 | 5.5794222 | 280.7732 |
| Toilet | Round4 | 468 | 894.1648 | 70.803745 | 4.9510079 | 222.5606 |
| Toilet | Round5 | 313 | 462.0417 | 32.586034 | 4.511038 | 116.2711 |

Table S2. QIIME mapping file for Sample 1 (sample accession number SAMEA4670724)

| #SampleID | BarcodeSequence | LinkerPrimerSequence | RoomType | Sampling | ReversePrimer | Description |
| --- | --- | --- | --- | --- | --- | --- |
| Main.Round1.M16 | TCACGTACTA | AGAGTTTGATCCTGGCTCAG | Main | Round1 | TGCTGCCTCCCGTAGGAGT | Floor dust from KBV main room floor, round 1 |
| Kitchen.Round1.M19 | TGTACTACTC | AGAGTTTGATCCTGGCTCAG | Kitchen | Round1 | TGCTGCCTCCCGTAGGAGT | Floor dust from KBV kitchen room floor, round 1 |
| Toilet.Round1.M20 | ACGACTACAG | AGAGTTTGATCCTGGCTCAG | Toilet | Round1 | TGCTGCCTCCCGTAGGAGT | Floor dust from KBV toilet room floor, round 1 |

Table S3. QIIME mapping file for Sample 2 (sample accession number SAMEA4670725)

| #SampleID | BarcodeSequence | LinkerPrimerSequence | RoomType | Sampling | ReversePrimer | Description |
| --- | --- | --- | --- | --- | --- | --- |
| Toilet.Round2.M16 | TCACGTACTA | AGAGTTTGATCCTGGCTCAG | Toilet | Round2 | TGCTGCCTCCCGTAGGAGT | Floor dust from KBV toilet room floor, round 2 |
| Main.Round2.M19 | TGTACTACTC | AGAGTTTGATCCTGGCTCAG | Main | Round2 | TGCTGCCTCCCGTAGGAGT | Floor dust from KBV main room floor, round 2 |
| Kitchen.Round2.M20 | ACGACTACAG | AGAGTTTGATCCTGGCTCAG | Kitchen | Round2 | TGCTGCCTCCCGTAGGAGT | Floor dust from KBV kitchen room floor, round 2 |

Table S4. QIIME mapping file for Sample 3 (sample accession number SAMEA4670726)

| #SampleID | BarcodeSequence | LinkerPrimerSequence | RoomType | Sampling | ReversePrimer | Description |
| --- | --- | --- | --- | --- | --- | --- |
| Kitchen.Round3.M16 | TCACGTACTA | AGAGTTTGATCCTGGCTCAG | Kitchen | Round3 | TGCTGCCTCCCGTAGGAGT | Floor dust from KBV kitchen room floor, round 3 |
| Toilet.Round3.M19 | TGTACTACTC | AGAGTTTGATCCTGGCTCAG | Toilet | Round3 | TGCTGCCTCCCGTAGGAGT | Floor dust from KBV toilet room floor, round 3 |
| Main.Round3.M20 | ACGACTACAG | AGAGTTTGATCCTGGCTCAG | Main | Round3 | TGCTGCCTCCCGTAGGAGT | Floor dust from KBV main room floor, round 3 |

Table S5. QIIME mapping file for Sample 4 (sample accession number SAMEA4670727)

| #SampleID | BarcodeSequence | LinkerPrimerSequence | RoomType | Sampling | ReversePrimer | Description |
| --- | --- | --- | --- | --- | --- | --- |
| Main.Round4.M16 | TCACGTACTA | AGAGTTTGATCCTGGCTCAG | Main | Round4 | TGCTGCCTCCCGTAGGAGT | Floor dust from KBV main room floor, round 4 |
| Toilet.Round4.M19 | TGTACTACTC | AGAGTTTGATCCTGGCTCAG | Toilet | Round4 | TGCTGCCTCCCGTAGGAGT | Floor dust from KBV toilet room floor, round 4 |
| Kitchen.Round4.M20 | ACGACTACAG | AGAGTTTGATCCTGGCTCAG | Kitchen | Round4 | TGCTGCCTCCCGTAGGAGT | Floor dust from KBV kitchen room floor, round 4 |

Table S6. QIIME mapping file for Sample 5 (sample accession number SAMEA4670728)

| #SampleID | BarcodeSequence | LinkerPrimerSequence | RoomType | Sampling | ReversePrimer | Description |
| --- | --- | --- | --- | --- | --- | --- |
| Kitchen.Round5.M16 | TCACGTACTA | AGAGTTTGATCCTGGCTCAG | Kitchen | Round5 | TGCTGCCTCCCGTAGGAGT | Floor dust from KBV kitchen room floor, round 5 |
| Main.Round5.M19 | TGTACTACTC | AGAGTTTGATCCTGGCTCAG | Main | Round5 | TGCTGCCTCCCGTAGGAGT | Floor dust from KBV main room floor, round 5 |
| Toilet.Round5.M20 | ACGACTACAG | AGAGTTTGATCCTGGCTCAG | Toilet | Round5 | TGCTGCCTCCCGTAGGAGT | Floor dust from KBV toilet room floor, round 5 |


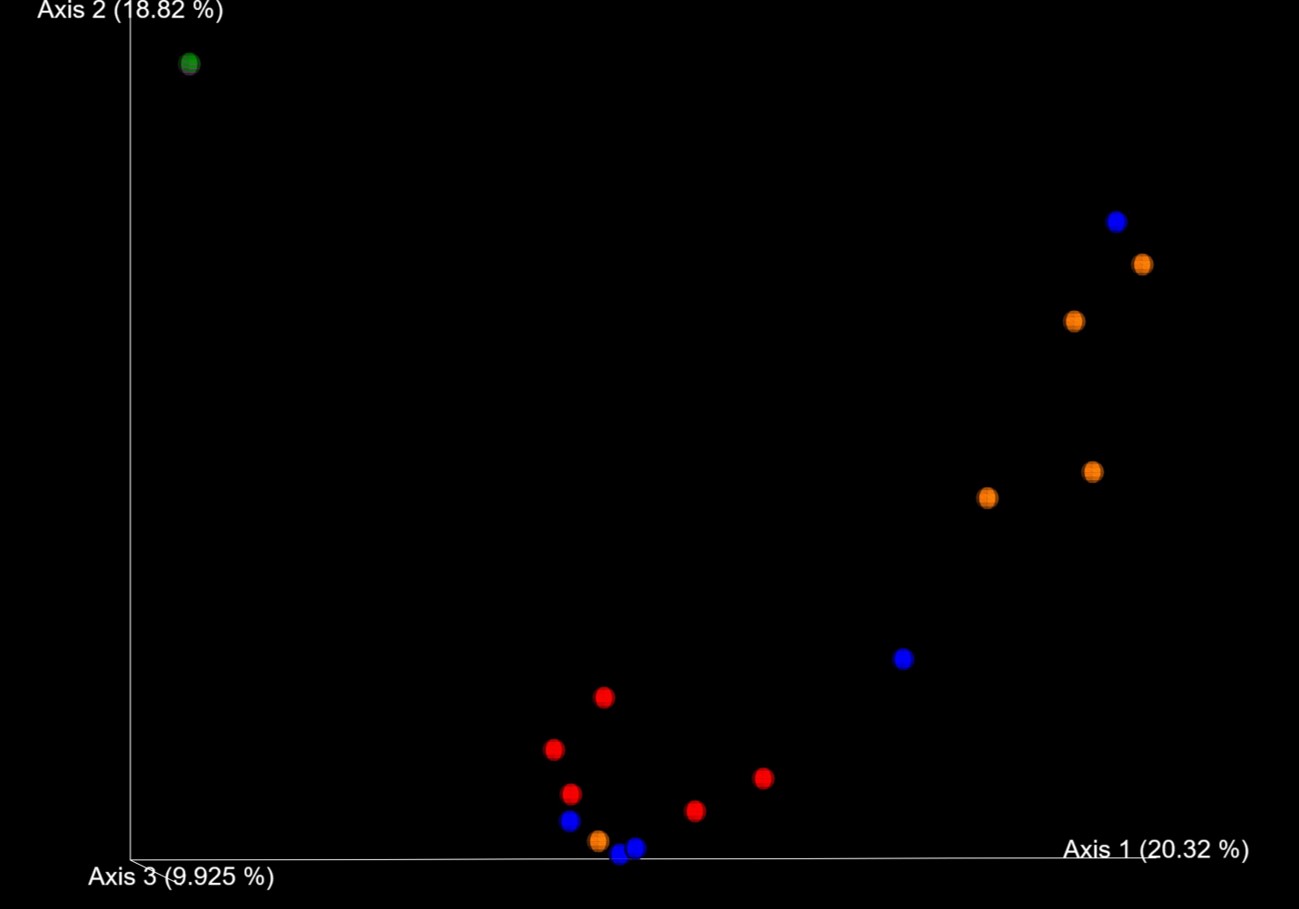


Figur S1. Principal Coordinate plot (PCoA) of Bray-Curtis distances plotting samples and negative controls. Red: toilet samples, blue: main room samples, orange: kitchen samples, green: extraction blank negative control, purple: PCR reaction blank negative control.


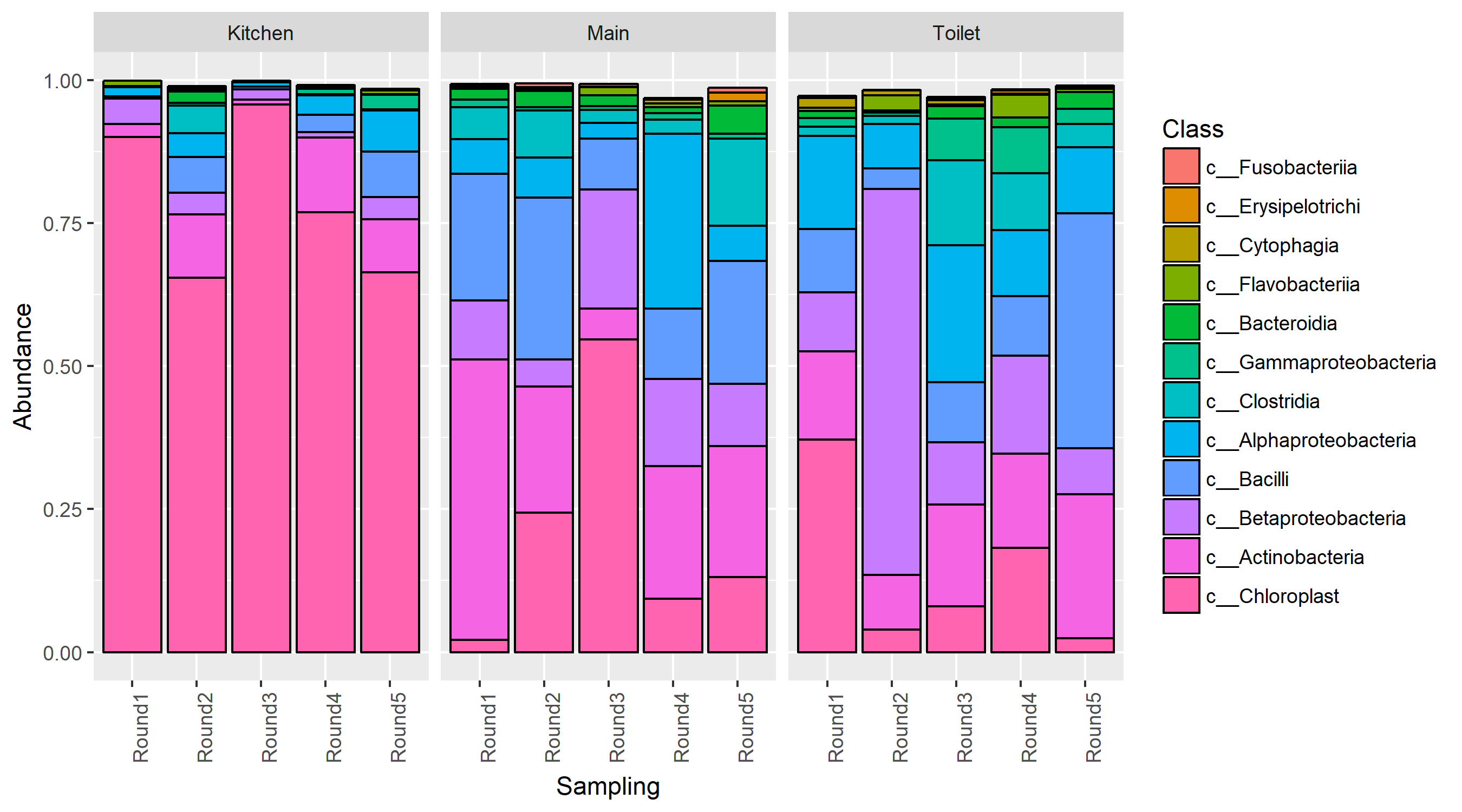


Figure S2. Changes in the bacterial composition at class level over time in the kindergarten, with chloroplast sequences retained
